# Identification of Compounds That Interfere with High‐Throughput Screening Assay Technologies

**DOI:** 10.1002/cmdc.201900395

**Published:** 2019-09-19

**Authors:** Laurianne David, Jarrod Walsh, Noé Sturm, Isabella Feierberg, J. Willem M. Nissink, Hongming Chen, Jürgen Bajorath, Ola Engkvist

**Affiliations:** ^1^ Hit Discovery, Discovery Sciences, R&D BioPharmaceuticals AstraZeneca Goteborg Pepparedsleden 1 431 83 Mölndal Sweden; ^2^ Department of Life Science Informatics, B-IT LIMES Program Unit Chemical Biology and Medicinal Chemistry Rheinische Friedrich-Wilhelms-Universität Bonn Endenicher Allee 19c 53115 Bonn Germany; ^3^ Hit Discovery, Discovery Sciences, R&D BioPharmaceuticals AstraZeneca Cambridge Alderley Park Macclesfield SK10 4TG UK; ^4^ Data Science and AI, Drug Safety & Metabolism, R&D BioPharmaceuticals AstraZeneca Gothenburg Pepparedsleden 1 431 83 Mölndal Sweden; ^5^ Hit Discovery, Discovery Sciences, R&D BioPharmaceuticals AstraZeneca Boston 35 Gatehouse Drive Waltham MA 02451 USA; ^6^ Computational Chemistry, Oncology R&D AstraZeneca Cambridge Science Park, Milton Road Cambridge CB4 0WG UK

**Keywords:** assay interference, computational chemistry, frequent hitters, high-throughput screening, machine learning

## Abstract

A significant challenge in high‐throughput screening (HTS) campaigns is the identification of assay technology interference compounds. A **C**ompound **I**nterfering with an **A**ssay **T**echnology (CIAT) gives false readouts in many assays. CIATs are often considered viable hits and investigated in follow‐up studies, thus impeding research and wasting resources. In this study, we developed a machine‐learning (ML) model to predict CIATs for three assay technologies. The model was trained on known CIATs and non‐CIATs (NCIATs) identified in artefact assays and described by their 2D structural descriptors. Usual methods identifying CIATs are based on statistical analysis of historical primary screening data and do not consider experimental assays identifying CIATs. Our results show successful prediction of CIATs for existing and novel compounds and provide a complementary and wider set of predicted CIATs compared to BSF, a published structure‐independent model, and to the PAINS substructural filters. Our analysis is an example of how well‐curated datasets can provide powerful predictive models despite their relatively small size.

## Graphical abstract

**Prediction of compounds** that interfere with HTS technology through a machine‐learning model: In this work, compounds that interfere with a high‐throughput screening technology are identified in counter‐screen assays and their chemical structures are used to train a random‐forest model to predict the behavior of new compounds. The model performs well, with respective ROC AUC values of 0.70, 0.62, and 0.57 in AlphaScreen, FRET, and TR‐FRET technologies and outperforms another published statistical method.

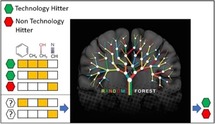

## Introduction

High‐throughput screening (HTS)^1^, ^2^ is a widely used approach in lead discovery. This method implies that activity data for hundreds of thousands of compounds can be generated in very little time. An important process in HTS is HTS triaging^3^, ^4^ in which hits are tested for their validity experimentally or statistically and are eliminated when exhibiting an undesirable behavior in one or multiple assays. Detection and removal of undesirable compounds serves to make follow‐up of hits more efficient, by not wasting resources on false hits. Compounds with undesirable behaviors can be of different categories.^5^ First, these include truly active but nonselective compounds that modulate multiple targets through the desired mechanism of action (MoA) (e.g., nonselective kinase ATP site binders) or compounds that engage the target through an undesirable MoA. Second, it includes compounds for which the assay measurement gives a false indication about the engagement of the target by the compound. In such occurrences, compounds can be described as false positive or false negative and their false readouts can be due to impurities, chemical reactivity or interference mechanisms with the assay technology. Herein, we introduce the term ′CIAT′ to refer to a **C**ompound that **I**nterferes with an **A**ssay **T**echnology. Such interference compounds are the focus of our study. Mechanisms of such interference vary^6^, ^7^ but include inhibition of secondary enzymes in coupled assays containing multiple enzymes, fluorescence quenching and compounds that interfere with an assay′s mechanism (e.g., biotin mimetics in bead‐based assays can compete for binding to the bead).

Several computational methods have been developed for the HTS triage process. Pearce et al.^8^ analyzed the promiscuity correlation of primary HTS data to a set of compound functional group and property filters. They observed a stronger correlation with functional group filters which decreased the number of compounds considered for triage by 12 %. A study from Baell et al.^9^ identified substructural motifs responsible for the promiscuous behavior of compounds by testing and analyzing a library of compounds at high concentration (25 and 50 μm) in a panel of assays using the AlphaScreen technology‐platform. This study led to the development of 480 substructure filters that are linked to assay interference and referred to as **P**an‐**A**ssay **In**terference Compound**s** (PAINS). PAINS filters have become popular for compounds triaging and are straightforward and easy to use. Over the years, the applicability of PAINS filters has been discussed and criticized.^5^, ^10^, ^11^ It has been reported that the presence of PAINS substructure does not necessarily imply an undesirable mechanism and the applicability domain of the PAINS is limited by the chemical space and the unique assay technology used in building the filters.

The BadApple^12^ service assigns a promiscuity score to compound structures based on their promiscuity, calculated from screening results and associated with their molecular scaffolds. While chemical scaffolds can be inherently promiscuous,^13^, ^14^ any specific pharmacophore or substructure can have a high impact on a compound's promiscuity.^15^, ^16^ The Hit Dexter platform^17^, ^18^ predicts various types of frequent‐hitters, which are compounds that are active in many assays, based on a set of highly tested PubChem compounds represented as molecular fingerprints. Hit Dexter 2.0 covers both primary and confirmatory dose–response assays and classifies compounds as promiscuous or not with an MCC of 0.64 and a ROC AUC of 0.96. A study from Ghosh et al.^19^ applied machine‐learning, pharmacophore analysis and molecular docking to flag false positive hits in Luciferase HTS assays. These three approaches yielded a balanced accuracy of 89.7, 74.2, and 67.2 % respectively.

In contrast to the above‐mentioned methods that make predictions based on chemical structure, the Binomial Survivor Function (BSF)^20^, ^21^ assesses the statistical probability that a primary screening result is not based on experimentally observed anomalous compound behavior (such as technology interference, aggregation or reactivity), given a combination of its screening results and the overall hit rates in historical screens. BSF is calculated by assuming that a screening is a binomial experiment. In HTS, compounds are tested in hundreds of assays and the likelihood that any compound will be a true active in an unbiased assay is small. Based on historical data, it is possible to estimate the likelihood of a compound to be active and to classify it as a frequent‐hitter or not. Thus, the BSF score is independent of chemical structure but is dependent on adequate annotation of the screening results. A downside of BSF is that it cannot be applied to predict the promiscuity of previously untested, or novel, compounds.

Herein, we present a novel random forest classification (RFC) model that predicts assay technology interference from molecular structures. We compare this model with the BSF score and the results from applying PAINS filters. The latter comparison is done to investigate the ability of the PAINS filters to identify CIATs. In contrast to methods which use HTS primary screening results as input data, the datasets used for the RFC models contain results from historical counter‐screen assays, which are used to experimentally rule out assay technology interference mechanisms from primary HTS datasets. To our knowledge, this is the first example of counter‐screen data being used to model assay technology interference in a systematic manner. Three extensively applied HTS assay technologies, AlphaScreen,^22^ FRET,^23^ (Förster resonance energy transfer) and TR‐FRET^24^ (time‐resolved fluorescence resonance energy transfer) are investigated.

## Results and Discussion

### Data collection

Data from primary single‐concentration HTS of three technologies (AlphaScreen, FRET, TR‐FRET) was collected from the HTS database at AstraZeneca. For compounds active in the primary assays, results from their corresponding down‐stream artefact assays were collected. An artefact assay (also called counter‐screen assay) contains all assay components except the target protein and is used for experimental triage. Compounds were classified into CIATs (compounds active in the artefact assay, thus assumed to be interfering with the assay technology) and NCIATs (compounds inactive in the artefact assay).

Most published computational promiscuity analysis methods rely on statistical analysis of historical activity data extracted from primary screens^17^, ^20^, ^25^ and, less frequently, from confirmatory concentration–response screens.^18^, ^26^ An unusually high hit rate for a compound across many screens for an HTS technology can be an indication that the compound is causing interference. Similarly, if a compound is active against a wide range of targets or target families, it must be treated carefully as it is likely unselective or perhaps prone to false readouts. An activity cutoff value of the promiscuity degree can be applied to flag a compound as abnormally promiscuous. A high degree of promiscuity can result from target interference by undesired MoA, assay technology interference (in which case the assay measurement would not reflect the true value) or a general problematic mechanism (reactivity, aggregation, solubility issues, impurities).

Herein, we focus on the use of artefact assays to detect and confirm promiscuity in compounds. The number of primary assays within each technology in the in‐house database as well as the number of primary assays associated with an artefact assay are shown in Table 1. In this study, we propose a predictive‐model that is built using artefact assay data and compare this model to a second one based on primary assay data only. To ensure consistency among the datasets for both models and to make a fair comparison of their performance, we only considered datapoints of primary assays for which artefact data was available, unless stated otherwise.

**Table 1:** Data collection.

Technology	Primary assay	Primary assay with artefact screen
AlphaScreen	69	8
FRET	249	21
TR‐FRET	114	7

### Application of PAINS filters

For each technology, PAINS filters were applied on defined CIATs and NCIATs. PAINs have been derived from a specific set of AlphaScreen data^9^ and their scope is therefore expected to be somewhat limited, especially when applied on other HTS technologies. We wanted to compare the PAINS filters performance across different technologies to analyze their applicably domain and because they are often applied as general compound filters irrespective of HTS technology, an approach that may remove good compounds from HTS datasets inadvertently. In general, PAINS filters had a very low accuracy when used to identify CIATs. The filters performed slightly better for the AlphaScreen dataset (9 % of CIATS correctly predicted) than for FRET and TR‐FRET (1.5 % of CIATs correctly predicted). The higher performance for AlphaScreen predictions can be explained by the fact that PAINS substructures were derived from an AlphaScreen‐based dataset. A higher correlation between CIATs and PAINs for AlphaScreen compared to other technologies was observed as well at Lilly Research Laboratories.^27^ However, even for AlphaScreen, the accuracy is quite low when the filters are applied to our data. A similar observation was made in a study by Capuzzi et al.^11^

One hypothesis may be that the relatively low accuracy is due to differences between our dataset and the one on which the filters were built, in terms of compound structures as well as screening conditions, such as compound concentration. These hypotheses could not be verified as the PAINS dataset was never publicly disclosed. It is also possible that specific PAINS substructures were classified as undesirable internally and were thus not part of the screening deck.^28^ PAINS filters are applicable to AlphaScreen data but their reassessment using greater structural diversity and screening conditions more similar to those commonly used would be beneficial.^10^

Ideally, PAINS‐like substructure filters should be derived separately for each HTS technology, in order to be as predictive as possible, and automatically updated when new data is uploaded into a screening database. It may be important to consider details such as the signal direction and emission wavelength of the readout. For instance, if the aim of a model is to predict compound interference by fluorescence quenching at a certain wavelength, it will need to be trained on screening data generated with fluorescence read‐out at the desired wavelength, where the fluorescence signal decreases to generate an active compound. Such a granular level of assay annotation is possible using, for example, the public BioAssay Ontology,^29^, ^30^ which could assist in creating promising datasets for modeling.

### Machine‐learning model development

A random forest classifier model (RFC) ^[31]^ was developed to discriminate between CIATs and NCIATs. For each technology, compounds experimentally validated as CIATs and NCIATs by their technology artefact screening results and represented as ECFP4 fingerprints constituted the training sets for RFC. Our model relies on experimental data, from artefact assays, enabling predictions based on compounds that were experimentally validated as interfering with an assay technology or not.

### Performance of the RFC model

An initial assessment of the RFC model performance was done using 10‐fold cross‐validation on the training sets, producing ROC AUC values across the assay technologies ranging from 0.72 to 0.83. To ensure that these performances were not due to overfitting, compounds class labels (CIAT and NCIAT) were randomly shuffled and cross‐validation was repeated. In this case, the best ROC AUC was 0.49, supporting the idea that the correlation between structural features and compound interference with assay technology can be modeled using our methodology, for all three assay technologies. The mean ROC AUC values are shown in Table 2.

**Table 2:** Performance of RFC in cross‐validation and label randomization.

Technology		Cross‐validation ROC AUC		Label randomization ROC AUC	
		Mean ROC range	Overall mean	Mean ROC range	Overall mean
AlphaScreen		0.79–0.81	0.80±0.033	0.48–0.49	0.49±0.017
FRET		0.73–0.75	0.74±0.056	0.48–0.49	0.48±0.021
TR‐FRET		0.72–0.78	0.76±0.05	0.48–0.49	0.49±0.012

For each assay of a technology, interference behavior of compounds was predicted based on the CIATs and NCIATs available in the other assays of the same technology. This was done to simulate the scenario of a prospective analysis in which all the assays available for a given technology would form the training set and be used to derive predictions for an external assay test set. The performance of the model was evaluated for each assay using multiple metrics. It should be noted that this study differs from many other machine‐learning studies in the sense that a compound can be present in both the training and the assay test set. This occurred for compounds tested in more than one primary screen. To distinguish between compounds tested more than once in a primary screen for a specific technology, the set was divided into two parts. Compounds tested more than once are referred to as Set A, and compounds found only in one assay as Set B. In such a situation, compounds in Set B correspond to novel compounds that have not been tested before in any of the used screens for the assay technology. For some assays, no set‐B compounds were available. These assays were therefore discarded for the analysis (unless stated otherwise), we considered 7, 10, and 6 primary assays for AlphaScreen, FRET, and TR‐FRET, respectively.

**Figure 1:**
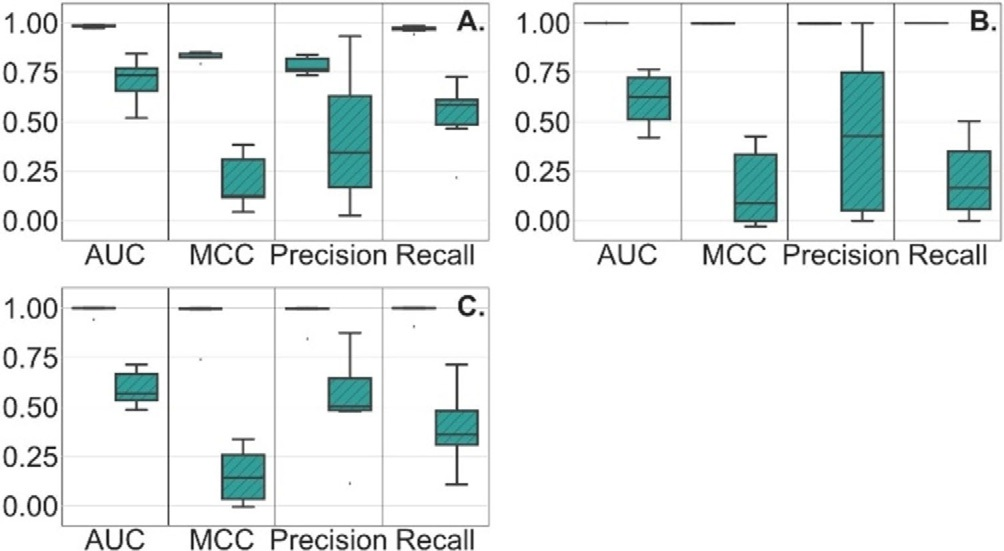
Performance of RFC for Set A (plain blue) and Set B (hashed) for AlphaScreen (A), FRET (B), and TR‐FRET (C).

For all the assay technologies, compounds in Set A were, as expected, well predicted by RFC with a recall, precision and ROC AUC ranging from 0.90 to 1.0, 0.73 to 1.0 and 0.94 to 1.0., respectively. For AlphaScreen, while the performance was close to perfect for Set A, it still made some erroneous predictions. An explanation may be that the structural differences between CIATs and NCIATs are not well captured by ECFP4, or compounds could be lacking some specific feature that may be important for the classification model. In Set B, CIATs were predicted by RFC with an average recall, precision and ROC AUC of 0.36, 0.46 and 0.63, respectively. The distribution of the metrics for both Sets A and B are reported in Figure 1.

### Comparison of performance between RFC, BSF, and PAINS

Because most existing tools used to identify CIATs are based on statistical analysis of compound activity across primary multiple assays, we compared the performance of our RFC model to BSF, which is an example of the former. The RFC model performance was also compared with the PAINS filters.

**Figure 2:**
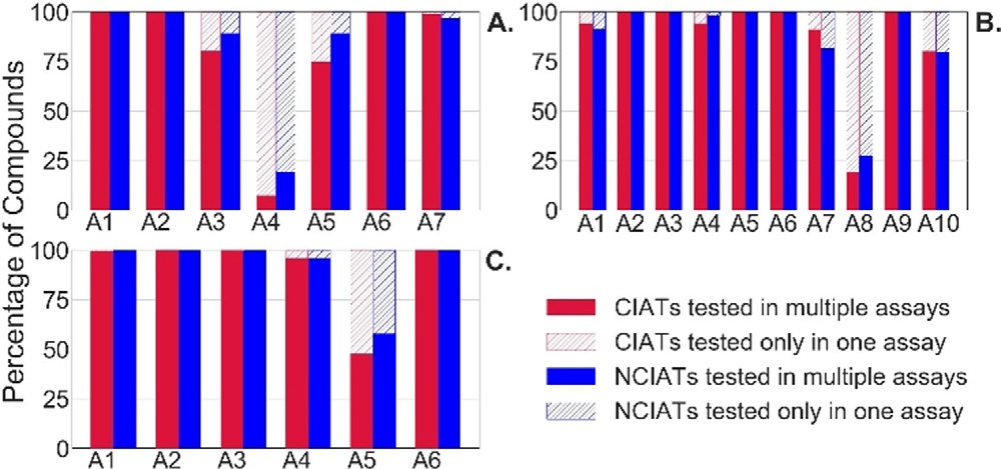
Percentage of CIATs and NCIATs tested in one or multiple assays (A1: assay 1, A2: assay 2, etc.) in AlphaScreen (A), FRET (B) and TR‐FRET (C). In a prospective scenario, compounds tested in multiple assays would be predicted by RFC and BSF, and compounds tested only in one assay would be predicted by RFC only.

As the BSF score is based on historical HTS data, its performance in identifying anomalous compounds increases with the amount of data available. However, one major drawback of BSF is that it is unable to do any prediction for compounds that have not been previously tested, which is something that RFC can do. To compare the performance of RFC to BSF, BSF score was calculated based on the primary assays that have been followed‐up by an artefact assay. BSF requires a compound to be tested at least once and preferably a high number of times to be correctly predicted. Figure 2 shows the percentage of compounds that could be predicted only by RFC, as BSF could not predict them, these compounds having been tested in only one assay. RFC is therefore more useful in the long run and especially on new technologies with very little data from primary assays.

**Figure 3:**
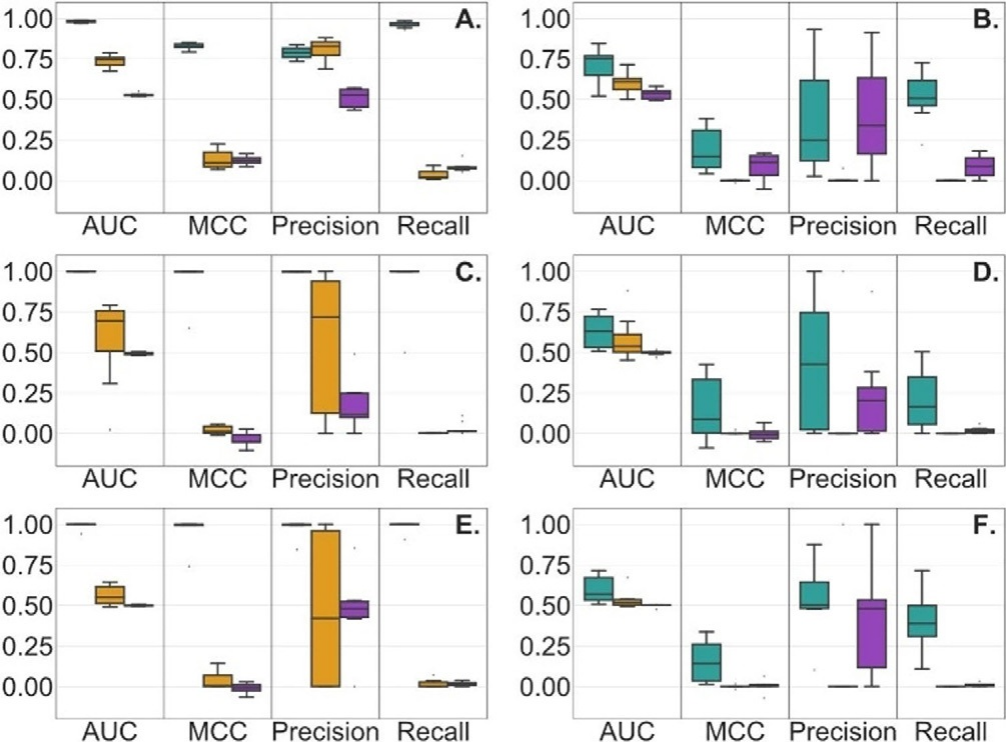
Performance of RFC (green), BSF (blue) and PAINS (violet) for AlphaScreen (A & B), FRET (C & D), and TR‐FRET (E & F). Here the performance is evaluated considering Set A (A, C, E) and Set B (B, D, F).

Figure 3 reports performance metrics for each of the assays of Sets A and B. RFC could predict the behavior of more compounds than BSF, therefore only compounds that could be predicted by both models were considered.

For Set A, compounds were predicted more accurately by RFC than BSF and PAINS, presenting a ROC AUC of 0.99 in average (0.63 for BSF and 0.50 for PAINS). This was expected as the compounds were present in both the RFC training and assay test sets. BSF performed better than PAINS in all three technologies. For the PAINS filters, the precisions were slightly better in the AlphaScreen technology (0.51 compared with 0.18 and 0.46 in FRET and TR‐FRET, respectively).

For Set B, RFC outperformed the other two models with a ROC AUC of 0.64 in average (0.57 for BSF and 0.51 for PAINS). RFC′s recalls were especially high (average of 0.35, 0.0001 and 0.04 for RFC, BSF and PAINS). It was observed that the precision of RFC and PAINS was similar in AlphaScreen, while it was higher for RFC in the other technologies. The detailed metrics for Set B are available as Supporting Information (Tables S1 and S2).

**Figure 4:**
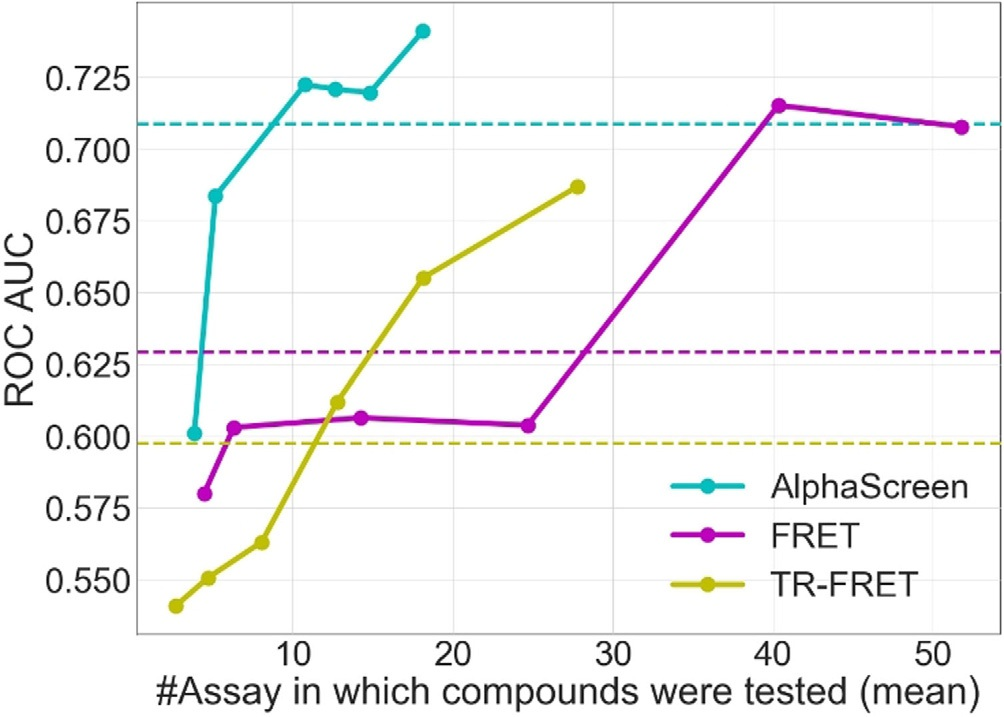
BSF performance for an increasing number of assays. BSF was calculated while considering primary assays with an artefact and then 20 %, 40 %, 60 %, 80 %, and 100 % of the assays. Curves show the performance of BSF, and the dashed lines represent the performance of RFC for the same dataset as BSF, for each technology. The performance metrics for BSF when considering 100 % of the assays are available as Supporting Information (Table S3).

As mentioned above, BSF performs better at identifying frequent‐hitters with large amounts of data. To have a full perspective on the RFC and BSF performances, we analyzed BSF′s performance while considering an increasing number of assays among all available primary screening results of a technology. For BSF to outperform RFC, compounds needed to be tested on average 10 times for AlphaScreen and TR‐FRET and 30 times for FRET as shown on Figure 4. RFC required fewer experiments to be done as a compound only needs to be tested once in an artefact assay to be validated as CIAT. RFC will be especially useful for new technologies for which very few primary assays are available.

### Behavior analysis of RFC

Among CIATs, true positives (CIATs correctly predicted as CIATs) and false negatives (CIATs wrongly predicted as NCIATs) were investigated for RFC to better understand the model's behavior.

**Figure 5:**
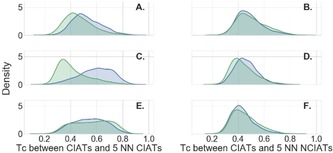
Density plots of the average distribution of Tc of true positive CIATs (blue) and false negative CIATs (green) in relation to their five nearest neighbors among CIATs (plots A, C, E) and NCIATs (plots B, D, F) in their corresponding training sets in RFC. Plots A and B, C and D, E and F correspond, respectively, to AlphaScreen, FRET, and TR‐FRET.

We investigated why CIATs are correctly or wrongly predicted by RFC. Tanimoto similarity coefficients (Tc) were computed between: 1) true positives and their five nearest neighbors (NN) among CIATs and NCIATs in the training set, in an all‐by‐all comparison, and 2) false negatives and their five NN among CIATs and NCIATs in the training set, in an all‐by‐all comparison. The average Tc was considered for each CIATs. Figure 5 shows the distribution of these average Tc values. For AlphaScreen, it was observed that true positives had a slightly higher similarity to training set CIATs in comparison with the false negatives. This pattern is even more pronounced in FRET, the distribution of Tc values being more shifted to the right for true positive, indicating a higher similarity of these compounds toward CIATs in the training set. In TR‐FRET technology, true positives and false negatives shared the same Tc distribution while compared to CIATs and NCIATs. However, RFC predictions were still sufficiently accurate, leading to the hypothesis that CIATs cannot be recognized by a simple similarity comparison. RFC can capture combinations of features inferring better predictions than similarity comparison.

### Application of CIATs filters or predictive models

**Figure 6:**
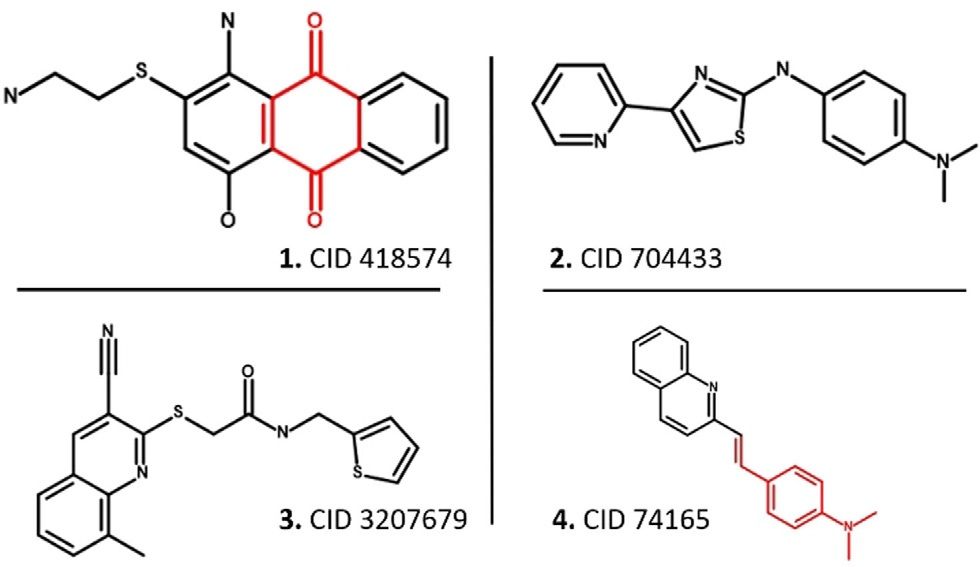
Example compounds with differential behavior with regard to assay technologies. PAINS substructures are shown in red. **1**. 415874 is an AlphaScreen‐CIAT, a FRET‐NCIAT, and a TR‐FRET‐NCIAT; it contains a PAIN substructure (quinone_A(370)). **2**. 704433 is an AlphaScreen‐CIAT and a FRET‐NCIAT. **3**. 3207679 is a TR‐FRET‐CIAT and an AlphaScreen‐NCIAT. **4**. 74165 is an AlphaScreen‐NCIAT, a FRET‐CIAT, and a TR‐FRET‐CIAT; it contains a PAIN substructure (anil_di_alk_B(251)).

We remind readers that CIATs are not necessarily compounds with no interest in drug discovery. They are simply compounds interfering with a specific HTS technology. CIATs filters or models can be applied prior to any experimental testing to guide the choice of HTS technology. PAIN‐compounds, which technically are CIATs according to the AlphaScreen dataset they were extracted from, are often removed prior to an analysis when they might have been valid hits had another technology been used. As an example we show four compounds which were CIATs in one of the technology we investigated and NCIAT in another in Figure 6. These compounds are common to our in‐house database and PubChem.

**Figure 7:**
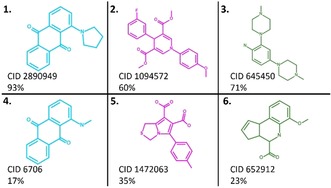
CIATs well identified (1, 2, 3) and misclassified (4, 5, 6) by RFC in AlphaScreen (blue), FRET (magenta), and TR‐FRET (green). The percent probability to be a CIAT according to RFC is annotated next to each molecule. Both AlphaScreen‐CIATs contain a PAIN substructure (quinone_A(370)), and a TRF‐CIAT (6) contains the PAIN substructure anil_alk_ene(51).

To explore the applicability of our RFC model, we applied it on compounds extracted from PubChem artefact assays. RFC identified 35 % of CIATs in average, with a mean ROC AUC and precision of 0.67 and 0.63 respectively. Details of the metrics and confusion matrixes are available in the Supporting Information (Tables S5–S11). Examples of CIATs that were recognized or misclassified by RFC are shown in Figure 7.

## Conclusions

Assay interference represents a major conundrum in drug discovery. Compounds that interfere with assay technologies, for example fluorescence quenchers or emitters, and reactive compounds, can obstruct the efficient identification of the most promising drug lead series of an HTS campaign. To identify such compounds, we have built a machine‐learning model, RFC, based on artefact assays. RFC performed well at predicting CIATs for three HTS technologies and required compounds from the training set to be tested only once in an artefact assay. When a large dataset is available (i.e., with many compounds tested in many assays), a statistical method such as the BSF score is efficient, however compounds need to be tested a significant number of times for the score to outperform RFC. Furthermore, contrary to BSF, RFC can predict the behavior of new compounds using only their structures and is therefore the only option for novel compounds. CIATs identified by the two methods are complementary and therefore projects can benefit from using RFC and BSF in tandem. The method applied in this study shows the importance, for computational models, of well‐annotated screening results related to full screening cascades (primary, artefact, orthogonal assays, etc.). The scarcity of well‐curated and structured HTS datasets in the public domain prevents efficient model building for scientists that do not have access to large, proprietary databases. We hope that this study will motivate the deposition of counter‐screen assay data and the curation of public databases based on HTS technologies to better annotate CIATs and decrease the amount of false hits that are investigated in follow‐up studies.

## Experimental Section

**Data collection and curation: AstraZeneca database**: Primary screening data associated to three different HTS technologies (AlphaScreen,^22^ FRET^23^ and TR‐FRET^24^) were collected from the AstraZeneca HTS database. These technologies are luminescent and fluorescent methods widely used in HTS. It is important to mention that each compound in our dataset was tested in primary assays and their corresponding artefact assays (also called counter‐screen, control or clean assays). A primary assay aims to test the activity of compounds on a target at a single concentration. An artefact screen consists of testing the same compounds without any target or with a significantly different target. All artefact screens considered in this study are concentration–response assays, which are more reliable than primary assays, and aim to identify CIATs.

In an assay, a compound can have multiple data points and activity flags. This can be due to the use of different assay plates, a follow‐up test at a different concentration or a solubility issue. As the main predictive model used in our study was a classification one, it was necessary for the compounds to have only one activity flag (active or inactive) per assay as well as per technology. In the primary assay files, two types of activity flags were available, one assigned by a normal assay score and based on percent effect, and a second assigned by a Z‐score.^32^ The first score considers the plate‐to‐plate variability and normalize the compounds measurements in relation to a control. The Z‐score method assumes that most compounds are inactive and uses them as control. Compounds measurements are rescaled considering the variation within the plate. The Z‐score flag was always the one considered in our study, except when it was not available. In the primary screening datasets, compounds that were found to be active and inactive at the same conditions were classified as active in primary assays. Compounds with inconclusive activity were discarded from further analysis.

Compound activities in artefact assays were determined based on concentration–response data. For each assay, an activity cutoff was assigned by screening experts considering a threshold concentration (based on IC_50_ or EC_50_) or a threshold percentage (based on percent inhibition).

**Training sets for classification models**: Compounds found to be active in a primary assay were classified as CIATs and NCIATs when active and inactive, respectively, in an artefact assay. Compound tested in multiple artefact assays, when considering one technology, and found to have different activity flags, were classified as active. CIATs and NCIATs constituted the training sets for the machine‐learning model. The training sets composition is annotated in Table 3.

**Table 3:** Training sets composition.

Technology		Training sets for RFC		
		Compounds	CIATs	NCIATs
AlphaScreen		68 540	15 330 (22.4 %)	53 210 (77.6 %)
FRET		32 966	7422 (22.5 %)	25 544 (77.5 %)
TR‐FRET		41 121	18 093 (44 %)	23 028 (56 %)

**Molecular representations**: For every compound classified as CIAT or NCIAT, ECFP4 fingerprints with 1024 bits and a fragment radius of 2 were generated using the Morgan circular fingerprint implemented in RDKit.^33^ Structures that could not be parsed by RDKit were discarded. Our in‐house collection is curated; thus, our datasets did not contain duplicate structures.

**Machine‐learning model**: A random forest classifier (RFC) is a classification algorithm combining ensemble tree‐structured classifiers.^31^ A typical RFC model is made up of hundreds of decision trees and the most important votes determine the final prediction.

The RFC model was implemented using SKLearn.^34^ In a prospective analysis, the training set of the model would contain all the CIATs and NCIATs previously identified in artefact assays. To mimic the condition of a prospective analysis, the training sets for a technology were built excluding one assay, this assay containing the compounds that we wish to predict. This process was repeated for each assay of a technology, thus taking into account the diversity of the datasets. Indeed, some assays can contain specific scaffolds or features that were not represented in other assays due to the target under investigation. Such over‐ or under‐representation of structures may impact the performance of the model. The compounds to be predicted are referred to as the assay test set. The process resulted in the prediction of two types of compounds to which we referred, respectively as Sets A and B: 1) Compounds that are available in both the training set and the assay test set, and 2) compounds that are available solely in the assay test set.

For each assay, the hyperparameters were optimized using a random‐search with a 3‐fold cross‐validation using two thirds of the compounds with known behavior as training set and one third as test set. Model performance was evaluated based on the average MCC obtained over all folds. The RFC hyperparameters that were tested and chosen are available in the Supporting Information (Table S4).

Some assays contained very few to none CIATs to be predicted. For meaningful performance evaluation, the analysis was restricted to assays with more than five CIATs, thus ensuring that the model performance would not be underappreciated because of some particularly complex structures.

**Performance measures**: To ensure that the RFC model's performance is based on an accurate structural distinction between CIATs and NCIATs, and not due to an overfitting issue, two methods were applied. First, the labels pertaining to compounds in the training set were randomly shuffled. If the model maintains a good performance with incorrect label, then the observed performance would be due to overfitting. Second, a tenfold cross‐validation was done on the test set, insuring that all the compounds are responsible of the predictions.

To assess model performances, four metrics were used, namely the area under the receiver operating characteristic (ROC) curve (AUC),^35^ Matthew's correlation coefficient (MCC), precision and recall.

The principal measure of the models is the MCC. It is a balanced measure of prediction quality which considers the true positive (TP), false positive (FP), true negative (TN) and false negative (FN). It covers a range from −1 to 1, with 1 indicating a perfect prediction, −1 a perfect inverse prediction, and 0 a random prediction [Equation (1)]:

(1) $$MCC=\;\frac{TP\;\times TN-FP\;\times FN}{\sqrt{\left(TP+FP)(TP+FN)(TN+FP)(TN+FN\right)}}$$

The AUC is a second measure of the performance of the mode. It shows how well a model was able to rank the compounds according to the probabilities given by the machine learning algorithm.

The recall, also called sensitivity, represents the proportion of real positive cases that are correctly predicted as such (in our analysis, it represents the proportion of CIAT correctly identified by a model). The precision is the proportion of true positive predictions that are correctly real positives [Equations (2), (3)]:

(2) $$Recall=\;\frac{TP}{TP+FN}$$

(3) $$Precision=\;\frac{TP}{TP+FP}$$

In this study, scripts were written in Python 3.6.5. Machine‐learning models were built using SKLearn 0.19.1^34^ as well as RDkit 2018.03.1.0.^33^

**Binomial survivor function score**: A score to characterize CIATs referred to as BSF^20^ was applied and the results compared with those obtained with RFC. This score is calculated for a compound by considering the number of times it was tested in an assay to the number of times it was active. The BSF score is a negative logarithm of a chance and represents the probability that the activity of a compounds is the result of randomness while considering the hit rate among the assays. If the activity is high (usually higher than 2, corresponding to 1 % chance that the compound is not a CIAT), it suggests that the observed activity is an outlier and is higher than expected. The cutoff applied to classify a compound as CIATs in this analysis is of pBSF≥2. Equation (4) describes the Binomial Survivor Function, in which *A* represents the number of screens in which a compound is active, *N* is the total number of screens in which a compound was tested, and *h* is the hit rate.

(4) pBSF=-10log(∑a=ANNaha1-hN-a

Unless stated otherwise, BSF was applied considering all the primary assays pertaining to a technology. Some technologies can be used to identify compounds with a biological activity on specific target families. For example, the ADP‐Glo technology has often been used to target kinases. In such cases, being based on historical activity data, BSF could classify a compound as being CIAT when it is instead a promiscuous compound with a true multi‐target activity or polypharmacology. The technologies investigated in this study targeted a wide range of proteins (39, 103 and 56 in AlphaScreen, FRET and TR‐FRET respectively pertaining to 2, 8, and 3 families, for example, enzyme, receptor, viral protein, etc.), and therefore BSF should only detect true CIATs interfering with the assay technology.

In a similar manner to RFC, BSF was applied to predict the compounds behaviors in one assay using the information pertaining to all the other assays. This was done for each assay of a technology.

**Pan‐assay interference compounds**: All the compounds investigated in this study were passed through PAINS filters,^9^ which included 480 substructures linked to assay interference encoded in SMARTS format.^36^

**Data collection and curation: PubChem**: CR artefact assays for AlphaScreen, FRET and TR‐FRET were collected from PubChem Bioassay.^37^ Assay AIDs and compositions are shown in Table 4. Active and inactive compounds were classified as CIATs and NCIATs, respectively and compounds with inconclusive activities were not considered further. Compounds which were CIATs and NCIATs depending of the assay were also discarded.

**Table 4:** Data collection from PubChem.

Technology	AID	CIAT	NCIAT
AlphaScreen	1730, 1159604, 720541	599	2025
FRET	435026	209	593
TR‐FRET	1641, 504689	149	412

**Availability of data and materials**: Our dataset could not be made available due to its proprietary nature. The source code of our predictive model (RFC) is available upon request.

## Conflict of interest

*The authors declare no conflict of interest*.

## Supporting information

As a service to our authors and readers, this journal provides supporting information supplied by the authors. Such materials are peer reviewed and may be re‐organized for online delivery, but are not copy‐edited or typeset. Technical support issues arising from supporting information (other than missing files) should be addressed to the authors.

Supplementary
