# Supplementary material for: Identification of Compounds That Interfere with High‐Throughput Screening Assay Technologies
Source: ChemMedChem. 2019 Sep 19;14(20):1795–802. doi: 10.1002/cmdc.201900395 (PMC6856845; doi:10.1002/cmdc.201900395)
Supplement: Supplementary file 1 — Supplementary [file CMDC-14-1795-s001.pdf]

CHEMMEDCHEM
Chemistry & Drug Discovery

Supporting Information

Identification of Compounds That Interfere with High-Throughput Screening Assay Technologies

Laurianne David,*[a, b] Jarrod Walsh,[c] Noé Sturm,[d] Isabella Feierberg,[e] J. Willem M. Nissink,[f] Hongming Chen,[a] Jürgen Bajorath,[b] and Ola Engkvist[a]

cmdc_201900395_sm_miscellaneous_information.pdf

SUPPLEMENTARY MATERIAL

Table S1. Confusion matrix and metrics for RFC applied to each assay of three technologies (Set B)

Technolo-gy	Assay	#False NCIATs	#False CIATs	#True NCIATs	#True CIATs	MCC	Precision	AUC	Recall	F1 score	Balanced accuracy
Alpha-Screen	A1	220	1932	7181	192	0,12	0,09	0,73	0,47	0,15	0,63
	A2	525	1415	1749	744	0,13	0,34	0,58	0,59	0,43	0,57
	A3	27	2690	10808	72	0,11	0,03	0,84	0,73	0,05	0,76
	A4	1190	416	1473	1889	0,38	0,82	0,77	0,61	0,7	0,7
	A5	2184	160	981	2234	0,3	0,93	0,77	0,51	0,66	0,68
	A6	79	67	314	22	0,04	0,25	0,52	0,22	0,23	0,52
	A7	384	766	2236	601	0,32	0,44	0,73	0,61	0,51	0,68
FRET	A1	169	38	817	5	-0,03	0,12	0,48	0,03	0,05	0,49
	A2	421	89	1085	38	0,01	0,3	0,52	0,08	0,13	0,5
	A3	786	113	1670	411	0,36	0,78	0,74	0,34	0,48	0,64
	A4	137	500	7403	8	0	0,02	0,58	0,06	0,02	0,5
	A5	626	9	68	532	0,17	0,98	0,74	0,46	0,63	0,67
	A6	199	161	472	202	0,25	0,56	0,67	0,5	0,53	0,62
	A7	157	356	5796	11	0,01	0,03	0,51	0,07	0,04	0,5
	A8	452	134	2027	247	0,37	0,65	0,69	0,35	0,46	0,65
	A9	6	0	16	2	0,43	1	0,77	0,25	0,4	0,63
	A10	5	13	595	0	-0,01	0	0,42	0	0	0,49
TRF	A1	850	404	1242	900	0,28	0,69	0,69	0,51	0,59	0,63
	A2	2	5	9	5	0,34	0,5	0,71	0,71	0,59	0,68
	A3	116	2	142	14	0,2	0,88	0,59	0,11	0,19	0,55
	A4	8841	3691	12606	3772	0,08	0,51	0,55	0,3	0,38	0,54
	A5	1039	589	1232	538	0,02	0,48	0,53	0,34	0,4	0,51
	A6	212	1021	1606	130	-0,01	0,11	0,49	0,38	0,17	0,5

Table S2. **Confusion matrix and metrics for BSF applied to each assay of three technologies (Set B)**

Technolo -gy	Assay	#False NCIATs	#False CIATs	#True NCIATs	#True CIATs	MCC	Precision	AUC	Recall	F1 score	Balanced accuracy
Alpha-Screen	A1	308	12	8099	1	0,008	0,077	0,564	0,003	0,006	0,501
	A2	93	1	607	0	-0,015	0,000	0,609	0,000	0,000	0,499
	A3	99	2	13496	0	-0,001	0,000	0,716	0,000	0,000	0,500
	A4	3072	0	1889	0	0,000	0,000	0,628	0,000	0,000	0,500
	A5	4416	0	1139	0	0,000	0,000	0,558	0,000	0,000	0,500
	A6	100	0	370	0	0,000	0,000	0,501	0,000	0,000	0,500
	A7	793	0	2672	0	0,000	0,000	0,631	0,000	0,000	0,500
FRET	A1	33	0	233	0	0,00	0,00	0,45	0,00	0,00	0,50
	A2	433	0	1074	0	0,00	0,00	0,52	0,00	0,00	0,50
	A3	1195	0	1782	1	0,02	1,00	0,57	0,00	0,00	0,50
	A4	132	1	6473	0	0,00	0,00	0,52	0,00	0,00	0,50
	A5	1157	0	77	0	0,00	0,00	0,69	0,00	0,00	0,50
	A6	401	0	633	0	0,00	0,00	0,50	0,00	0,00	0,50
	A7	168	0	6152	0	0,00	0,00	0,88	0,00	0,00	0,50
	A8	656	0	2122	0	0,00	0,00	0,55	0,00	0,00	0,50
	A9	8	0	16	0	0,00	0,00	0,63	0,00	0,00	0,50
	A10	4	0	485	0	0,00	0,00	0,48	0,00	0,00	0,50
TRF	A1	1677	1	1578	0	-0,018	0,000	0,673	0,000	0,000	0,500
	A2	7	0	14	0	0,000	0,000	0,500	0,000	0,000	0,500
	A3	129	0	144	0	0,000	0,000	0,539	0,000	0,000	0,500
	A4	12605	0	16297	6	0,016	1,000	0,513	0,000	0,001	0,500
	A5	1571	0	1817	0	0,000	0,000	0,525	0,000	0,000	0,500
	A6	163	0	1521	0	0,000	0,000	0,495	0,000	0,000	0,500

Table S3. **Confusion matrix and metrics for BSF applied to each assay of three technologies when considering all the primary assays available in a technology (Set B)**

Technolo-gy	Assay	#False NCIATs	#False CIATs	#True NCIATs	#True CIATs	MCC	Precision	AUC	Recall	F1 score	Balanced accuracy
**Alpha-Screen**	A1	228	143	7968	81	0,29	0,36	0,92	0,26	0,30	0,62
	A2	70	14	594	23	0,34	0,62	0,84	0,25	0,35	0,61
	A3	68	181	13317	31	0,21	0,15	0,81	0,31	0,20	0,65
	A4	2816	10	1879	256	0,17	0,96	0,79	0,08	0,15	0,54
	A5	4356	3	1136	60	0,04	0,95	0,55	0,01	0,03	0,51
	A6	98	1	369	2	0,09	0,67	0,60	0,02	0,04	0,51
	A7	766	11	2661	27	0,12	0,71	0,67	0,03	0,06	0,51
**FRET**	A1	27	24	209	6	0,08	0,20	0,49	0,18	0,19	0,54
	A2	375	41	1033	58	0,17	0,59	0,72	0,13	0,22	0,55
	A3	1095	12	1770	101	0,20	0,89	0,87	0,08	0,15	0,54
	A4	114	526	5948	18	0,03	0,03	0,67	0,14	0,05	0,53
	A5	1134	0	77	23	0,04	1,00	0,63	0,02	0,04	0,51
	A6	387	7	626	14	0,08	0,67	0,65	0,03	0,07	0,51
	A7	143	112	6040	25	0,14	0,18	0,78	0,15	0,16	0,57
	A8	483	85	2037	173	0,33	0,67	0,85	0,26	0,38	0,61
	A9	8	0	16	0	0,00	0,00	0,82	0,00	0,00	0,50
	A10	4	4	481	0	-0,01	0,00	0,60	0,00	0,00	0,50
**TRF**	A1	1658	1	1578	19	0,07	0,95	0,79	0,01	0,02	0,51
	A2	7	0	14	0	0,00	0,00	0,55	0,00	0,00	0,50
	A3	129	0	144	0	0,00	0,00	0,67	0,00	0,00	0,50
	A4	10889	141	16156	1722	0,26	0,92	0,88	0,14	0,24	0,56
	A5	1509	10	1807	62	0,12	0,86	0,85	0,04	0,08	0,52
	A6	161	43	1478	2	-0,03	0,04	0,38	0,01	0,02	0,49

Table S4. **Hyperparameters tested by Randomized Search**

Parameter	Option
Number of trees in random forest (n_estimators)[a]	**10**,50,100,150,200,300,400
Maximum fraction of features considered at every split (max_features)[a]	**Sqrt**, 0.2, 0.4, 0.6, 0.8, None
Weight associated with class (class_weight)[a]	**None**, balanced
Minimum number of samples required to be an internal node (min_samples_split)[a]	**2**, 5, 10
Minimum number of samples required to be a leaf node (min_samples_leaf)[a]	**1**, 5, 10
Maximum number of levels in trees (max_depth)[a]	**None**, 10, 12, 14, 16, 18, 20, 50, 100
Method of selecting samples for training each tree (bootstrap)[a]	**True**, False

[a] Parameter name in the scikit-learn implementation
Bold options indicate the default values

The hyperparameters selected for each technology are the following:

Hyperparameters for AlphaScreen:

- n_estimators = 100, class_weight='balanced', max_features='sqrt', min_samples_leaf=5, min_samples_split=5, max_depth=None, bootstrap=False

Hyperparameters for FRET (2 possibilities depending of the assay):

- n_estimators = 100, class_weight=None, max_features='sqrt', min_samples_leaf=5, min_samples_split=5, max_depth=None, bootstrap=False
- n_estimators = 150, class_weight='balanced', max_features='sqrt', min_samples_leaf=1, min_samples_split=2, max_depth=50, bootstrap=False

Hyperparameters for TRF (2 possibilities depending of the assay):

- n_estimators = 200, class_weight='balanced', max_features='sqrt', min_samples_leaf=5, min_samples_split=2, max_depth=None, bootstrap=True
- n_estimators = 150, class_weight='balanced', max_features='sqrt', min_samples_leaf=1, min_samples_split=2, max_depth=50, bootstrap=False

Application of RFC on PubChem Assays.

Table S5. **Performance Metrics.**

Technology	AID	AUC	Recall	Precision	MCC	F1
AlphaScreen	1730	0.86	0.5	1.0	0.68	0.66
	1159604	0.74	0.27	0.4	0.17	0.32
	720541	0.48	0.15	0.74	0.18	0.25
FRET	435026	0.78	0.35	0.86	0.47	0.49
TR-FRET	1641	0.59	0.5	0.49	0.11	0.5
	504689	0.61	0.34	0.27	0.15	0.3

Table S6. **Confusion matrix for AID 1730 (AlphaScreen)**

AID 1730		Reality	
		CIAT	NCIAT
Prediction	CIAT	5	0
	NCIAT	5	57

Table S7. **Confusion matrix for AID 1159604 (AlphaScreen)**

AID 1159604		Reality	
		CIAT	NCIAT
Prediction	CIAT	14	21
	NCIAT	37	143

Table S8. **Confusion matrix for AID 720541 (AlphaScreen)**

AID 720541		Reality	
		CIAT	NCIAT
Prediction	CIAT	62	21
	NCIAT	345	431

Table S9. **Confusion matrix for AID 435026 (FRET)**

AID 435026		Reality	
		CIAT	NCIAT
Prediction	CIAT	72	12
	NCIAT	136	581

Table S10. **Confusion matrix for AID 1641 (TR-FRET)**

AID 1641		Reality	
		CIAT	NCIAT
Prediction	CIAT	45	46
	NCIAT	45	72

Table S11. Confusion matrix for AID 504689 (TR-FRET)

**<u>AID 504689</u>**		Reality	
		CIAT	NCIAT
Prediction	CIAT	20	53
	NCIAT	39	241
